# Clinical Associations and Prognostic Value of MRI-Visible Perivascular Spaces in Patients With Ischemic Stroke or TIA: A Pooled Analysis

**DOI:** 10.1212/WNL.0000000000207795

**Published:** 2023-12-13

**Authors:** Jonathan G. Best, Gareth Ambler, Duncan Wilson, Houwei Du, Keon-Joo Lee, Jae-Sung Lim, Kay Cheong Teo, Henry Mak, Young Dae Kim, Tae-Jin Song, Derya Selcuk Demirelli, Masashi Nishihara, Masaaki Yoshikawa, Marta Kubacka, Annaelle Zietz, Rustam Al-Shahi Salman, Hans Rolf Jäger, Gregory Y.H. Lip, Leonidas Panos, Martina B. Goeldlin, Lee-Anne Slater, Christopher Charles Karayiannis, Thanh G. Phan, Maximilian Bellut, Jill Abrigo, Cyrus Cheng, Thomas W. Leung, Winnie Chu, Francesca Chappell, Stephen D.J. Makin, Dianne H.K. van Dam-Nolen, M. Eline Kooi, Sebastian Köhler, Julie Staals, Grégory Kuchcinski, Régis Bordet, Florian Dubost, Joanna M. Wardlaw, Yannie O.Y. Soo, Felix Fluri, Velandai K. Srikanth, Simon Jung, Nils Peters, Hideo Hara, Yusuke Yakushiji, Dilek Necioglu Orken, Ji-Hoe Heo, Gary Kui Kai Lau, Hee-Joon Bae, David J. Werring

**Affiliations:** From the UCL Stroke Research Centre (J.G.B., D.W., D.J.W.) and Neuroradiological Academic Unit (H.R.J.), Department of Brain Repair and Rehabilitation, UCL Queen Square Institute of Neurology, London; Department of Statistical Science (G.A.), University College London, United Kingdom; Stroke Research Centre (H.D.), Department of Neurology, Fujian Medical University Union Hospital, Fuzhou, People's Republic of China; Department of Neurology (K.-J.L.), Korea University Guro Hospital, Seoul; Department of Neurology (J.-S.L.), Asan Medical Center, University of Ulsan College of Medicine, Seoul, Republic of Korea; Division of Neurology, Department of Medicine (K.C.T., G.K.K.L.) and Department of Diagnostic Radiology (H.M.), The University of Hong Kong; Department of Neurology (Y.D.K., J.-H.H.), Yonsei University College of Medicine, Seoul; Department of Neurology (T.-J.S.), Seoul Hospital, Ewha Womans University College of Medicine, Seoul, Republic of Korea; Department of Neurology (D.S.D.), Sisli Hamidiye Etfal Teaching and Research Hospital, University of Health Sciences, Turkey; Department of Radiology (M.N.), Saga University Faculty of Medicine; Division of Neurology (M.Y., H.H.), Department of Internal Medicine, Saga University Faculty of Medicine, Japan; Stroke Center Klinik Hirslanden Zürich (M.K., N.P.); University of Basel (M.K.); Department of Neurology and Stroke Center University Hospital and University of Basel (A.Z.), Switzerland; Centre for Clinical Brain Sciences (R.A.-S.S., F.C., J.M.W.), School of Clinical Sciences, University of Edinburgh; Liverpool Centre for Cardiovascular Science at University of Liverpool (G.Y.H.L.), Liverpool John Moores University and Liverpool Heart & Chest Hospital, United Kingdom; Department of Neurology (L.P., M.B.G., S.J.), Inselspital, Bern University Hospital, Switzerland; Department of Imaging, School of Clinical Sciences at Monash Health (L.-A.S.), Peninsula Clinical School, Peninsula Health (C.C.K.), and Stroke and Ageing Research Group, School of Clinical Sciences at Monash Health (T.G.P.), Monash University, Melbourne, Australia; Department of Neurology (M.B., F.F.), University Hospital of Würzburg, Germany; Department of Imaging and Interventional Radiology (J.A., W.C.) and Division of Neurology, Department of Medicine and Therapeutics (C.C., T.W.L., Y.O.Y.S.), Prince of Wales Hospital, The Chinese University of Hong Kong; Institute of Applied Health Sciences (S.D.J.M.), University of Aberdeen, United Kingdom; Biomedical Imaging Group Rotterdam (F.D.), Department of Radiology and Nuclear Medicine (D.H.K.D.-N.), Erasmus University Medical Centre, Rotterdam, The Netherlands; Departments of Radiology and Nuclear Medicine (M.E.K.) and Neurology (J.S.), CARIM School for Cardiovascular Diseases, Maastricht University Medical Centre; Department of Psychiatry and Neuropsychology (S.K.), School for Mental Health and Neuroscience, Maastricht University, The Netherlands; University of Lille (G.K., R.B.), Inserm, CHU de Lille, Lille Neuroscience & Cognition, France; National Centre for Healthy Ageing (V.K.S.), Peninsula Clinical School, Central Clinical School, Monash University, Melbourne, Australia; Department of Neurology (Y.Y.), Kansai Medical University, Osaka, Japan; Department of Neurology (D.N.O.), Istanbul Arel University, Turkey; and Department of Neurology (H.-J.B.), Seoul National University Bundang Hospital, Seoul National University College of Medicine, Seongnam, Republic of Korea.

## Abstract

**Background and Objectives:**

Visible perivascular spaces are an MRI marker of cerebral small vessel disease and might predict future stroke. However, results from existing studies vary. We aimed to clarify this through a large collaborative multicenter analysis.

**Methods:**

We pooled individual patient data from a consortium of prospective cohort studies. Participants had recent ischemic stroke or transient ischemic attack (TIA), underwent baseline MRI, and were followed up for ischemic stroke and symptomatic intracranial hemorrhage (ICH). Perivascular spaces in the basal ganglia (BGPVS) and perivascular spaces in the centrum semiovale (CSOPVS) were rated locally using a validated visual scale. We investigated clinical and radiologic associations cross-sectionally using multinomial logistic regression and prospective associations with ischemic stroke and ICH using Cox regression.

**Results:**

We included 7,778 participants (mean age 70.6 years; 42.7% female) from 16 studies, followed up for a median of 1.44 years. Eighty ICH and 424 ischemic strokes occurred. BGPVS were associated with increasing age, hypertension, previous ischemic stroke, previous ICH, lacunes, cerebral microbleeds, and white matter hyperintensities. CSOPVS showed consistently weaker associations. Prospectively, after adjusting for potential confounders including cerebral microbleeds, increasing BGPVS burden was independently associated with future ischemic stroke (versus 0–10 BGPVS, 11–20 BGPVS: HR 1.19, 95% CI 0.93–1.53; 21+ BGPVS: HR 1.50, 95% CI 1.10–2.06; *p* = 0.040). Higher BGPVS burden was associated with increased ICH risk in univariable analysis, but not in adjusted analyses. CSOPVS were not significantly associated with either outcome.

**Discussion:**

In patients with ischemic stroke or TIA, increasing BGPVS burden is associated with more severe cerebral small vessel disease and higher ischemic stroke risk. Neither BGPVS nor CSOPVS were independently associated with future ICH.

## Introduction

Visible perivascular spaces (PVS) are linear or ovoid structures visible on MRI of the brain, with similar intensity to the CSF.^1^ They indicate enlargement of the compartment between penetrating cerebral blood vessels and the surrounding glia limitans, thought to be a route for interstitial fluid exchange in cerebral homeostasis.^2^ Accumulating evidence links PVS to cerebrovascular disease. In cross-sectional studies of patients with previous ischemic stroke, PVS have been associated with increasing age, vascular risk factors, lacunar stroke subtype, lacunes, and white matter hyperintensities (WMH).^3-8^ Similar associations have been found in older adults without previous stroke.^9-11^ In patients with previous intracerebral hemorrhage, PVS within the basal ganglia region (BGPVS) have been linked to deep intracerebral hemorrhage and deep cerebral microbleeds and PVS within the deep white matter of the centrum semiovale (CSOPVS) to lobar intracerebral hemorrhage, cortical superficial siderosis, and strictly lobar cerebral microbleeds.^12-15^ PVS might therefore be a marker of cerebral small vessel disease (CSVD) and of the specific underlying small arteriopathy (deep perforator arteriopathy/hypertensive arteriosclerosis or cerebral amyloid angiopathy). However, the results of individual studies have varied, possibly reflecting small sample sizes and differences in PVS rating methods.^16^

Of importance, few studies have examined the prognostic significance of PVS. In a pooled analysis of 2 studies comprising 2,002 participants with previous ischemic stroke or transient ischemic attack (TIA), the presence of more than 20 BGPVS in the cerebral hemisphere with the highest burden was associated with incident ischemic stroke but not intracerebral hemorrhage after adjustment for vascular risk factors.^17^ In a multicenter study of 1,490 patients with atrial fibrillation (AF) initiating anticoagulation after ischemic stroke or TIA, the presence of more than 10 BGPVS in a single hemisphere was associated with both ischemic stroke and intracranial hemorrhage (ICH), adjusted for vascular risk factors and other MRI markers of CSVD.^18,19^ In older adults without previous stroke, higher BGPVS counts have been found to be associated with incident intracerebral hemorrhage, again adjusted for vascular risk factors and CSVD markers, but not ischemic stroke,^20^ and in a separate study, with all-cause vascular events (including stroke) and vascular mortality.^21^ Finally, in patients with cerebral amyloid angiopathy and previous intracerebral hemorrhage, the presence of more than 20 CSOPVS in a single hemisphere was associated with recurrent lobar intracerebral hemorrhage.^22^

Given these varied findings, we aimed to clarify the clinical associations and prognostic significance of PVS in patients with previous ischemic stroke or TIA—a high-risk population in which the risk of recurrent stroke is of particular interest, and MRI is often used—through a pooled analysis of individual patient data from prospective cohort studies. Based on previous studies not restricted to ICH survivors, our main hypothesis was that BGPVS would be independently associated with the risks of both ICH and ischemic stroke.

## Methods

We identified participants through the Microbleeds International Collaborative Network (MICON), a consortium of 38 prospective cohort studies that enrolled participants with previous ischemic stroke or TIA, obtained baseline MRI including sequences sensitive to paramagnetic susceptibility (enabling detection of cerebral microbleeds, necessary for a comprehensive assessment of CSVD), and followed up participants for at least 3 months for ischemic stroke, symptomatic ICH, or a composite of both. The studies contributing to MICON were identified through a systematic review and existing collaborations including METACOHORTS^23^ and STRIVE^1^ and screened for quality and risk of bias. The full details of this have been published.^24^ Studies that obtained baseline axial T2-weighted imaging were eligible for inclusion in the current analysis. Although PVS can be rated using other MRI sequences, including T1-weighted imaging,^9^ we excluded studies without axial T2 to obtain comparable ratings that could be pooled directly.

Each study collected baseline and follow-up data according to local protocols. For our pooled analysis, we prespecified our clinical variables of interest, based on clinical relevance and availability, as follows: age, hypertension, diabetes, hyperlipidemia, AF, previous ischemic stroke before the index event, previous intracerebral hemorrhage, and baseline antithrombotic use (none, antiplatelet only, vitamin K antagonist, or direct oral anticoagulant). Patients taking an antiplatelet and an anticoagulant were assigned to the relevant anticoagulant category. Our radiologic variables of interest were BGPVS, CSOPVS, cerebral microbleeds, WMH, lacunes, and cerebral atrophy. Our outcomes of interest were ischemic stroke (excluding TIA) and nontraumatic symptomatic ICH within 5 years of study enrollment. Outcomes were adjudicated locally.

Imaging ratings were performed locally. PVS were rated separately in the basal ganglia and centrum semiovale using a validated 5-level scale,^25^ which categorizes PVS burden as 0, 1–10, 11–20, 21–40, and more than 40 PVS, using the highest count from a single hemisphere and a single MRI slice. All raters were trained using a standardized manual.^26^ Because we expected that few participants would receive PVS ratings of 0 or more than 40, we combined the bottom 2 and top 2 categories for analysis, giving a 3-level variable corresponding to 0–10, 11–20, and 21 or more PVS. WMH were rated using the Fazekas scale,^27^ apart from one study that used the Age-Related White Matter Changes (ARWMC) scale.^28^ We defined a moderate-to-severe WMH burden as a score of 2 or 3 on the ARWMC or Fazekas scale, taking the highest available value from deep white matter or periventricular regions. Cerebral microbleeds were counted in lobar and nonlobar (infratentorial and deep supratentorial) regions. For analysis, we classified cerebral microbleeds as present or absent and cerebral microbleed distribution as strictly lobar, strictly nonlobar, or mixed. Lacunes were recorded as present or absent. Cerebral atrophy was quantified using the 4-point simplified Pasquier scale or equivalent for global cortical atrophy.^29^ We defined moderate-to-severe cerebral atrophy as a rating of 2 or 3. All thresholds for categorization were specified before analysis.

For statistical analysis, we pooled data from all participating studies to make a single dataset. We excluded study participants who lacked follow-up data or who had inadequate MRI for assessment of PVS burden. We investigated the clinical and radiologic associations of BGPVS and CSOPVS cross-sectionally, using multinomial logistic regression, with PVS burden as the dependent variable, estimating relative risk ratios. We accounted for clustering by including study as a random effect.^30^ We did not use ordinal logistic regression because the proportional odds assumption was violated for many variables. We also assessed the correlation between BGPVS and CSOPVS burden using the Spearman Rho. Next, we investigated prospective associations between PVS burden and stroke risk using Cox regression with a shared frailty term. We tested initially for univariable associations between PVS burden in each region and each outcome of interest. For each outcome, we then fitted a multivariable model containing BGPVS burden, CSOPVS burden, and all other clinical and radiologic variables with an association at *p* < 0.2 in univariable analysis. Although candidate predictors had already been selected on clinical grounds, we used variable selection to reduce the risk of overfitting, while choosing a lenient threshold to avoid omitting potentially important predictors. We plotted Kaplan-Meier estimates of the cumulative incidence of ischemic stroke and symptomatic ICH to 5 years from study enrollment, according to PVS burden. We handled missing data in our regression analyses using multiple imputation with chained equations (5 imputations) and checked the proportional hazards assumption using tests of scaled Schoenfeld residuals.

As a sensitivity analysis, and to quantify heterogeneity between studies, we performed a 2-stage random-effects meta-analysis for each outcome using an inverse variance model, with PVS burden in each region categorized as 0–10, 11–20, and 21 or more. We used a common confounder model with coefficients estimated in our main (pooled) dataset to adjust for the same variables included in the multivariable models used in our main analysis. Our statistical analysis used Stata version 17.

### Standard Protocol Approvals, Registrations, and Patient Consents

The MICON study was approved by the UK Health Research Authority (8/HRA/0188) and registered on PROSPERO (CRD42016036602). Included cohorts obtained ethical and regulatory approvals according to local requirements. Only fully anonymized data were shared, so that individual consent was not required for this pooled analysis.

### Data Availability

Requests for access to anonymized study data may be directed to the corresponding author. Approval by the study steering committee and the principal investigator of each cohort in the study will be required before data are shared.

## Results

**Figure 1:**
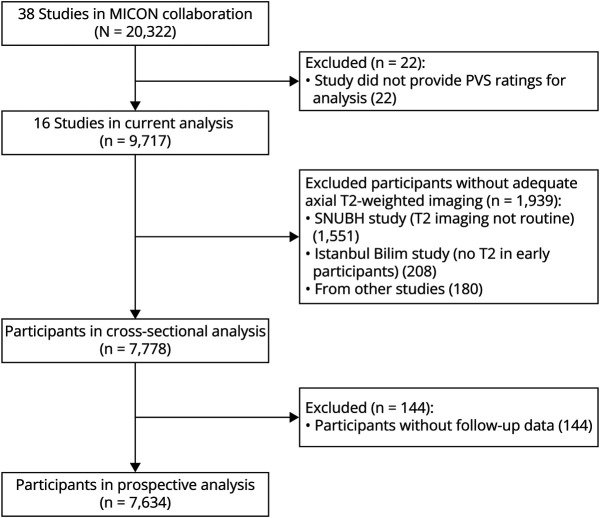
Study Flowchart

16 studies contributed to the current analysis (Figure 1). Most noncontributing studies did not acquire axial T2-weighted imaging or lacked resources to rate PVS. We excluded 1,939 participants from contributing studies because of missing or poor-quality axial T2-weighted imaging (mainly from 2 studies, SNUBH and Istanbul Bilim, in which T2-weighted imaging was optional), leaving a final study sample of 7,778 participants. Table 1 compares the baseline characteristics of included and excluded participants. The prevalence of AF and anticoagulant use was lower in excluded participants, reflecting the low prevalence of AF in the SNUBH study from which the largest number of participants were excluded. eTable 1 (links.lww.com/WNL/D239) summarizes baseline characteristics by study. Overall, participants were older, with a high prevalence of vascular risk factors and radiologic evidence of cerebral small vessel disease. Most of the participants had an ischemic stroke as their qualifying event, rather than TIA. Approximately one half had atrial fibrillation, and a similar proportion was prescribed oral anticoagulants. Very few participants had previous symptomatic ICH. Of the included studies, 9 were based in Europe and the Middle East, 6 in East Asia, and 1 in Australia.

**Table 1:** Baseline Characteristics of Included and Excluded Participants

Variable	Included (n = 7,778)	Excluded (n = 1,939)
Age (y)	70.6 (12.6)	68.1 (13.1)
Female sex	3,324/7,778 (42.7)	798/1,939 (41.2)
Atrial fibrillation	3,879/7,702 (50.4)	588/1,930 (30.5)
Hypertension	5,439/7,750 (70.2)	1,359/1,939 (70.1)
Diabetes	1888/7,505 (25.2)	550/1,939 (28.4)
Hyperlipidemia	2,912/7,360 (39.6)	632/1,935 (32.7)
Ischemic stroke before index event	1,161/7,749 (15.0)	272/1,939 (14.0)
Previous intracranial hemorrhage	121/7,398 (1.6)	23/1,904 (1.2)
Presentation with ischemic stroke	7,011/7,778 (90.1)	1,688/1,939 (87.1)
Antithrombotic use		
Antiplatelet only	3,655/7,776 (47.0)	1,194/1,939 (61.6)
VKA	2,475/7,776 (31.8)	476/1,939 (24.5)
DOAC	1,323/7,776 (17.0)	171/1,939 (8.8)
CMB presence	2,485/7,778 (31.9)	607/1,939 (31.3)
WMH score ≥2	3,609/7,764 (46.5)	939/1,654 (56.8)
BGPVS burden		
0–10	4,751/7,778 (61.1)	—
11–20	2016/7,778 (25.9)	—
21 +	1,011/7,778 (13.0)	—
CSOPVS burden		
0–10	3,212/7,778 (41.3)	—
11–20	2,545/7,778 (32.7)	—
21+	2021/7,778 (26.0)	—

Abbreviations: BGPVS = basal ganglia enlarged perivascular spaces; CMB = cerebral microbleed; CSOPVS = centrum semiovale enlarged perivascular spaces; DOAC = direct oral anticoagulant; VKA = vitamin K antagonist.

Values shown are prevalence (%) or mean (SD).

Across all included participants, 25.9% had 11–20 BGPVS in the more severely affected hemisphere, and 13.0% had 21 or more. CSOPVS counts were generally higher, with 32.7% of participants having 11–20 CSOPVS, and 26.0% having 21 or more. The distribution of CSOPVS scores varied more widely between studies than BGPVS scores (eTable 2, links.lww.com/WNL/D240). BGPVS and CSOPVS scores were moderately correlated (Spearman Rho = 0.40, *p* < 0.0001). Patients with very high PVS burdens were rare (41 or more BGPVS: 3.3%; 41 or more CSOPVS: 3.6%).

Table 2 summarizes baseline characteristics of participants according to BGPVS burden. Clinical characteristics associated with higher BGPVS burdens included increasing age, hypertension, a history of ischemic stroke before the index ischemic stroke or TIA, and previous intracranial hemorrhage. Radiologically, cerebral microbleed presence, moderate-to-severe WMH burden, lacune presence, and moderate-to-severe cerebral atrophy were all associated with higher BGPVS burdens, but strictly lobar cerebral microbleed presence and multiple strictly lobar cerebral microbleeds (suggestive of cerebral amyloid angiopathy) were not. We observed similar associations for increasing CSOPVS burden (Table 3), but these associations were consistently weaker. Previous ICH was not associated with CSOPVS burden nor were strictly lobar cerebral microbleed presence or multiple strictly lobar cerebral microbleeds. Both BGPVS and CSOPVS burdens were lower in patients with AF, who were on average older (mean age (SD) 75.0 (13.1) vs 66.4 (10.3) years, *p* < 0.0001) but had a lower burden of other CSVD markers than those without AF (moderate-to-severe WMH prevalence 42.9% vs 50.4%, *p* < 0.0001; CMB presence 29.1% vs 35.1%, *p* < 0.0001).

**Table 2:** Association of Baseline Characteristics With BGPVS Burden

Variable	0–10 BGPVS (n = 4,751)	11–20 BGPVS (n = 2,016)	21+ BGPVS (n = 1,011)	RRR: 11–20 BGPVS	RRR: 21+ BGPVS	*p* Value
Age (y)	67.9 (13.2)	73.8 (10.5)	77.1 (8.8)	1.05 (1.04–1.05)	1.09 (1.08–1.09)	<0.0001
Female sex	2,006/4,751 (42.2)	845/2,016 (41.9)	473/1,011 (46.8)	1.06 (0.95–1.18)	1.29 (1.12–1.49)	0.0025
Atrial fibrillation	2,593/4,702 (55.1)	815/2,000 (40.8)	471/1,000 (47.1)	0.66 (0.58–0.74)	0.84 (0.70–1.00)	<0.0001
Hypertension	3,057/4,734 (64.6)	1,559/2,013 (77.4)	823/1,003 (82.1)	2.04 (1.80–2.31)	2.60 (2.17–3.11)	<0.0001
Diabetes	1,103/4,610 (23.9)	549/1,948 (28.2)	236/947 (24.9)	1.25 (1.11–1.42)	0.99 (0.83–1.17)	0.0009
Hyperlipidemia	1753/4,545 (38.6)	763/1,898 (40.2)	396/917 (43.2)	1.02 (0.91–1.14)	1.05 (0.90–1.22)	0.83
Ischemic stroke before index event	618/4,729 (13.1)	330/2,012 (16.4)	213/1,008 (21.1)	1.47 (1.26–1.71)	1.88 (1.56–2.26)	<0.0001
Previous ICH	45/4,489 (1.0)	46/1,937 (2.4)	30/972 (3.1)	2.62 (1.70–4.02)	3.60 (2.19–5.92)	<0.0001
Presentation with ischemic stroke	4,221/4,751 (88.8)	1859/2,016 (92.2)	931/1,011 (92.1)	1.44 (1.18–1.79)	1.30 (1.00–1.70)	0.0006
CMB presence	1,204/4,751 (25.3)	759/2,016 (37.6)	522/1,011 (51.6)	1.86 (1.66–2.09)	3.52 (3.04–4.09)	<0.0001
Strictly deep CMBs	438/4,751 (9.2)	280/2,016 (13.9)	158/1,011 (15.6)	1.62 (1.37–1.91)	2.01 (1.63–2.48)	<0.0001
Mixed CMBs	284/4,751 (6.0)	265/2,015 (13.2)	249/1,010 (24.7)	2.51 (2.10–3.01)	5.67 (4.65–6.90)	<0.0001
Strictly lobar CMBs	482/4,751 (10.1)	214/2,016 (10.6)	115/1,011 (11.4)	1.07 (0.90–1.28)	1.13 (0.90–1.42)	0.49
Multiple strictly lobar CMBs	151/4,751 (3.2)	77/2,016 (3.8)	43/1,011 (4.3)	1.25 (0.94–1.67)	1.35 (0.94–1.93)	0.14
WMH score ≥2	1,532/4,741 (32.3)	1,257/2,014 (62.4)	820/1,009 (81.3)	3.81 (3.40–4.27)	10.3 (8.57–12.3)	<0.0001
Lacune presence	746/3,410 (21.9)	558/1,545 (36.1)	359/729 (49.2)	2.01 (1.75–2.30)	3.53 (2.96–4.20)	<0.0001
GCA score ≥2	545/2,953 (18.5)	455/1,299 (35.0)	323/630 (51.3)	2.32 (2.00–2.71)	4.07 (3.36–4.93)	<0.0001
CSOPVS: 0–10	2,609/4,751 (54.9)	454/2,016 (22.5)	149/1,011 (14.7)	—	—	<0.0001
CSOPVS: 11–20	1,465/4,751 (30.8)	776/2,016 (38.5)	304/1,011 (30.1)	2.86 (2.48–3.29)	4.37 (3.48–5.48)	
CSOPVS: 21+	677/4,751 (14.2)	786/2,016 (39.0)	558/1,011 (55.2)	5.90 (5.06–6.89)	15.1 (12.0–18.9)	

Abbreviations: BGPVS = basal ganglia enlarged perivascular spaces; CMB = cerebral microbleed; CSOPVS = centrum semiovale basal ganglia perivascular spaces; GCA = global cortical atrophy; ICH = intracranial hemorrhage; RRR = relative risk ratio; WMH = white matter hyperintensities.

Columns 2–4 show mean (standard deviation) for continuous variables and prevalence (%) for categorical variables.

An RRR >1 for age indicates that older patients are more likely to be in the corresponding BGPVS category than younger patients. An RRR >1 for a categorical variable (e.g., hypertension) indicates that patients with that characteristic are more likely to be in the corresponding BGPVS group than those without that characteristic.

**Table 3:** Association of Baseline Characteristics With CSOPVS Burden

Variable	0–10 CSOPVS (n = 3,212)	11–20 CSOPVS (n = 2,545)	21+ CSOPVS (n = 2021)	RRR: 11–20 CSOPVS	RRR: 21+ CSOPVS	*p* Value
Age (y)	71.0 (13.0)	69.6 (12.8)	71.4 (11.4)	1.00 (0.99–1.00)	1.01 (1.00–1.01)	<0.0001
Female sex	1,472/3,212 (45.8)	1,036/2,545 (40.7)	816/2,021 (40.4)	0.90 (0.81–1.01)	0.90 (0.80–1.02)	0.13
Atrial fibrillation	2,192/3,174 (69.1)	989/2,522 (39.2)	698/2,006 (34.8)	0.38 (0.33–0.43)	0.34 (0.30–0.40)	<0.0001
Hypertension	2,172/3,197 (67.9)	1764/2,537 (69.5)	1,503/2,016 (74.6)	1.33 (1.18–1.50)	1.69 (1.48–1.94)	<0.0001
Diabetes	735/3,113 (23.6)	618/2,460 (25.1)	535/1,932 (27.7)	1.11 (0.98–1.27)	1.24 (1.07–1.43)	0.014
Hyperlipidemia	1,251/3,067 (40.8)	906/2,401 (37.7)	755/1,892 (39.9)	0.86 (0.76–0.97)	0.89 (0.78–1.01)	0.037
Ischemic stroke before index event	525/3,195 (16.4)	333/2,534 (13.1)	303/2,020 (15.0)	0.99 (0.84–1.16)	1.14 (0.95–1.35)	0.23
Previous ICH	46/3,040 (1.5)	41/2,405 (1.7)	34/1,953 (1.7)	1.49 (0.92–2.40)	1.43 (0.84–2.43)	0.23
Presentation with ischemic stroke	2,854/3,212 (88.9)	2,312/2,545 (90.8)	1845/2,021 (91.3)	1.05 (0.86–1.27)	1.08 (0.87–1.34)	0.79
CMB presence	884/3,212 (27.5)	829/2,545 (32.6)	772/2,021 (38.2)	1.34 (1.18–1.51)	1.79 (1.57–2.05)	<0.0001
Strictly deep CMBs	278/3,212 (8.7)	292/2,545 (11.5)	306/2,021 (15.1)	1.36 (1.13–1.64)	2.02 (1.66–2.45)	<0.0001
Mixed CMBs	267/3,212 (8.3)	269/2,545 (10.6)	262/2,019 (13.0)	1.37 (1.14–1.32)	1.73 (1.42–2.12)	<0.0001
Strictly lobar CMBs	339/3,212 (10.6)	268/2,545 (10.5)	204/2,021 (10.1)	1.08 (0.90–1.29)	1.04 (0.85–1.28)	0.72
Multiple strictly lobar CMBs	122/3,212 (3.8)	92/2,545 (3.6)	57/2,021 (2.8)	1.06 (0.79–1.43)	0.82 (0.58–1.16)	0.33
WMH score ≥2	1,195/3,201 (37.3)	1,209/2,544 (47.5)	1,205/2,019 (59.7)	1.74 (1.55–1.95)	2.76 (2.43–3.14)	<0.0001
Lacune presence	463/1,993 (23.2)	626/2,019 (31.0)	574/1,672 (34.3)	1.46 (1.26–1.69)	1.63 (1.40–1.91)	<0.0001
GCA score ≥2	391/1,780 (22.0)	406/1,585 (25.6)	526/1,517 (34.7)	1.23 (1.03–1.46)	1.70 (1.43–2.03)	<0.0001

Abbreviations: BGPVS = basal ganglia enlarged perivascular spaces; CMB = cerebral microbleed; CSOPVS = centrum semiovale basal ganglia perivascular spaces; GCA = global cortical atrophy; ICH = intracranial hemorrhage; RRR = relative risk ratio; WMH = white matter hyperintensities.

Columns 2–4 show mean (standard deviation) for continuous variables and prevalence (%) for categorical variables.

**Figure 2:**
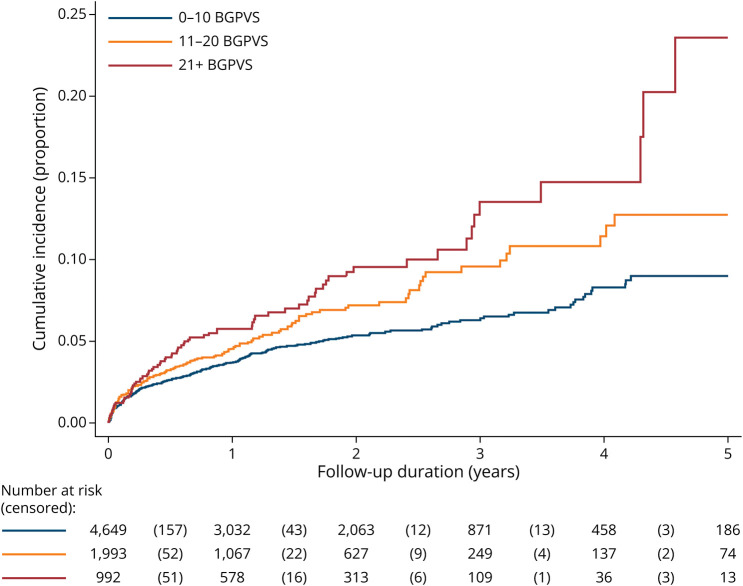
Cumulative Incidence of Ischemic Stroke According to BGPVS Burden BGPVS = basal ganglia enlarged perivascular spaces.

Follow-up information was available for 7,634 participants. The median follow-up duration was 1.27 years (interquartile range [IQR] 0.94–2.34) for ICH and 1.24 years (IQR 0.93–2.31) for ischemic stroke, over which 80 symptomatic ICH and 424 ischemic strokes were reported. Of the 80 ICH events, 71 were intracerebral, 8 subdural, and 1 a convexity subarachnoid hemorrhage. Of the 39 intracerebral hemorrhages for which detailed location was available, 20 were deep supratentorial, 10 infratentorial, 8 lobar, and 1 simultaneous lobar and deep. We imputed 418/7,778 (5.4%) observations for hyperlipidemia, 380/7,778 (4.9%) for previous ICH, 273/7,778 (3.5%) for diabetes, and <1% for all other variables. We omitted lacune presence and cerebral atrophy from our main regression analyses because of low availability (missing observations: 2,094/7,778 (26.9%) for lacune presence; 2,896 (37.2%) for cerebral atrophy). In univariable analysis, BGPVS burden was associated with the risks of ischemic stroke and ICH (Tables 4 and 5), with the hazard increasing with BGPVS burden. Multivariable analysis confirmed an independent association between BGPVS burden and ischemic stroke risk but did not show an association between BGPVS and ICH risk. Figure 2 and eFigure 1 (links.lww.com/WNL/D233) show the cumulative incidence of ischemic stroke and ICH according to BGPVS burden. We found no evidence of an association between CSOPVS and either outcome. The proportional hazards assumption held for all variables for both outcomes.

**Table 4:** Univariable and Multivariable Regression Results for Ischemic Stroke

Variable	Univariable HR (95% CI)	*p* Value	Multivariable HR (95% CI)	*p* Value
Age (y)	1.02 (1.01–1.03)	<0.001	1.01 (1.00–1.02)	0.18
Female sex	1.21 (1.00–1.46)	0.056	1.16 (0.95–1.41)	0.14
Atrial fibrillation	1.32 (0.97–1.80)	0.054	1.24 (0.81–1.92)	0.32
Hypertension	1.23 (0.98–1.53)	0.068	0.99 (0.78–1.24)	0.88
Diabetes	1.28 (1.02–1.61)	0.036	1.19 (0.95–1.51)	0.13
Hyperlipidemia	1.22 (0.98–1.50)	0.069	1.15 (0.92–1.43)	0.22
Ischemic stroke before index event	2.04 (1.63–2.56)	<0.001	1.81 (1.43–2.28)	<0.001
Previous intracranial hemorrhage	1.64 (0.90–2.99)	0.11	1.26 (0.68–2.32)	0.46
Antithrombotic use				
AP only	0.41 (0.26–0.62)	<0.001	0.47 (0.30–0.73)	0.0042
VKA	0.47 (0.29–0.74)		0.46 (0.28–0.75)	
DOAC	0.45 (0.27–0.76)		0.44 (0.25–0.76)	
CMB presence	1.24 (1.01–1.52)	0.040	1.06 (0.86–1.31)	0.58
WMH score ≥2	1.40 (1.15–1.70)	0.001	1.07 (0.86–1.35)	0.53
BGPVS				
11–20	1.36 (1.09–1.72)	<0.001	1.19 (0.93–1.53)	0.040
21+	1.86 (1.42–2.45)		1.50 (1.10–2.06)	
CSOPVS				
11–20	1.11 (0.87–1.41)	0.43	1.02 (0.80–1.31)	0.97
21+	1.20 (0.91–1.58)		0.99 (0.74–1.34)	

Abbreviations: AP = antiplatelet; BGPVS = basal ganglia enlarged perivascular spaces; CMB = cerebral microbleed; CSOPVS = centrum semiovale enlarged perivascular spaces; DOAC = direct oral anticoagulant; HR = hazard ratio; WMH = white matter hyperintensities.

**Table 5:** Univariable and Multivariable Regression Results for Symptomatic ICH

Variable	Univariable HR (95% CI)	*p* Value	Multivariable HR (95% CI)	*p* Value
Age (y)	1.04 (1.01–1.06)	0.001	1.02 (1.00–1.04)	0.12
Female sex	1.16 (0.75–1.80)	0.51	—	—
Atrial fibrillation	2.28 (1.27–4.11)	0.006	1.61 (0.59–4.41)	0.36
Hypertension	1.69 (0.98–2.94)	0.061	1.19 (0.68–2.10)	0.55
Diabetes	1.14 (0.69–1.86)	0.61	—	—
Hyperlipidemia	0.96 (0.60–1.54)	0.87	—	—
Ischemic stroke before index event	2.03 (1.21–3.38)	0.007	1.54 (0.91–2.59)	0.11
Previous intracranial hemorrhage	5.60 (2.56–12.23)	<0.001	3.81 (1.69–8.58)	0.001
Antithrombotic use				
AP only	0.44 (0.13–1.46)	0.006	0.78 (0.22–2.76)	0.19
VKA	1.22 (0.36–4.11)		1.28 (0.34–4.86)	
DOAC	0.56 (0.15–2.11)		0.60 (0.14–2.53)	
CMB presence	2.94 (1.83–4.73)	<0.001	2.47 (1.50–4.06)	<0.001
WMH score ≥2	2.06 (1.30–3.25)	0.002	1.28 (0.76–2.13)	0.35
BGPVS				
11–20	1.56 (0.93–2.61)	0.046	1.08 (0.62–1.89)	0.96
21+	2.05 (1.11–3.80)		1.03 (0.51–2.09)	
CSOPVS				
11–20	1.04 (0.60–1.81)	0.49	1.06 (0.60–1.86)	0.79
21+	1.41 (0.78–2.55)		1.24 (0.66–2.33)	

Abbreviations: AP = antiplatelet; BGPVS = basal ganglia enlarged perivascular spaces; CMB = cerebral microbleed; CSOPVS = centrum semiovale enlarged perivascular spaces; DOAC = direct oral anticoagulant; WMH = white matter hyperintensities.

In a 2-stage meta-analysis, the estimates for the association between BGPVS and ischemic stroke risk were similar to those from our pooled analysis, though confidence intervals were wider and estimates not statistically significant. Heterogeneity was low between studies (eFigure 2, links.lww.com/WNL/D234). We found no overall evidence of an association between BGPVS and ICH, though heterogeneity between studies was high (eFigure 3, links.lww.com/WNL/D235), driven primarily by a strong association between 11–20 BGPVS and ICH in the CROMIS-2 study. Despite using a common confounder model, both meta-analyses omitted some studies included in our pooled analysis because of quasi-complete separation or the absence of outcome events. As in our pooled analysis, we found no evidence for an association between CSOPVS and either outcome, with generally low heterogeneity between studies (eFigures 4 and 5, links.lww.com/WNL/D236 and links.lww.com/WNL/D237).

### Sensitivity Analyses

First, we repeated our Cox regression analyses as a complete case analysis, finding a similar association between BGPVS and ischemic stroke, though with a slightly weaker and nonsignificant *p* value reflecting reduced sample size (11–20 BGPVS: HR 1.21, 95% CI 0.93–1.58; for 21+ BGPVS: HR 1.48, 95% CI 1.06–2.09; *p* = 0.068).

Second, we repeated our analyses using multiple imputation to include lacune presence and global cortical atrophy as covariates. We found a similar but slightly weaker association between BGPVS and ischemic stroke (11–20 BGPVS: HR 1.19, 95% CI 0.92–1.53; for 21+ BGPVS: HR 1.48, 95% CI 1.07–2.03; *p* = 0.054), with no independent association between lacune presence or global cortical atrophy and ICH or ischemic stroke.

Third, because we observed variability in PVS counts between centers, we repeated our analyses omitting centers at which the proportion of participants receiving a rating of 21+ BGPVS was more than double the proportion for the whole study sample and then omitting those at which the proportion of participants receiving a rating of 21+ BGPVS was less than half the proportion for the whole study sample. We then did the same for CSOPVS. All results were similar (*detailed results not shown*).

Fourth, because our multivariable model for ICH included a relatively large number of variables for the number of ICH events observed, we tested the effect of adjusting for each covariate individually. The univariable association between BGPVS and ICH was substantially attenuated by adjustment for any of the following: age, cerebral microbleed presence, or WMH burden (eTable 3, links.lww.com/WNL/D241).

Finally, we tested whether our results were affected by more detailed parameterization of other CSVD markers in our multivariable model. The association between BGPVS and ischemic stroke was essentially unchanged by representing WMH burden as a 4-level ordinal variable or by recategorizing CMB burden as 0, 1, 2–4, and 5 or more (detailed results not shown).

### Predictive Value

Having found a significant association between BGPVS burden and ischemic stroke risk, we assessed the incremental predictive value of adding BGPVS burden to a Cox regression model comprising the CHA_2_DS_2_-VASc score using multiple imputation to account for missing observations for congestive heart failure (49.1% of observations imputed) and peripheral or coronary artery disease (48.5% of observations imputed) in some cohorts. We quantified discrimination through Harrell c-index, and generated confidence intervals for the difference in c-index using bootstrapping (200 iterations). In the whole study sample (including patients with and without AF), BGPVS burden significantly improved model fit (*p* = 0.0022) and slightly improved discrimination (c-index with BGPVS burden: 0.56; without, 0.55; difference in c-index 0.014, 95% CI −0.0030 to 0.031). In patients with AF, we observed significantly improved model fit (*p* = 0.0013) and a modest improvement in discrimination (c-index with BGPVS burden: 0.60; without, 0.58; difference in c-index 0.020, 95% CI −0.0060 to 0.046).

## Discussion

Our main finding is that BGPVS are strongly associated with vascular risk factors and established markers of CSVD in patients with previous ischemic stroke or TIA and are independently associated with the risk of future ischemic stroke, with the risk increasing with BGPVS burden. Although the mechanisms linking BGPVS to cerebrovascular disease remains unclear, perivascular space enlargement might occur in the setting of CSVD due to changes in vascular permeability, inflammation, or altered perivascular fluid flow secondary to changes in arterial compliance and pulsatility.^31-34^ We also found an increased risk of incident ICH in patients with higher BGPVS burdens, but no independent association in multivariable analysis. Overall, our findings suggest that considering BGPVS burden might contribute to ischemic stroke risk stratification, although in this study, we found only a small improvement in discrimination compared with the CHA_2_DS_2_-VASc clinical risk score, principally in participants with AF. Considering BGPVS burden is unlikely to add to the prediction of ICH over cerebral microbleeds and previous ICH, both of which were incorporated into the previously published MICON-ICH risk score.^35^

Our findings differ from those of the CROMIS-2 and 3C-Dijon studies (the former included in our pooled analysis), which found independent associations between higher BGPVS burdens and incident ICH.^18,20^ We considered whether this could be a subgroup effect because CROMIS-2 recruited only patients with AF initiating anticoagulation, but we did not find a similar association in other participating studies that recruited only patients with AF. The 3C-Dijon study recruited patients without previous stroke, and high BGPVS counts might be rarer and more significant in this lower risk population. However, another notable difference is that its results were not adjusted for cerebral microbleed presence, which we found to be the most important radiologic predictor of ICH and was associated with BGPVS. Whereas PVS might be an early feature of CSVD, cerebral microbleeds indicate vascular fragility and more advanced CSVD, more directly linked to ICH.

Despite the correlation between BGPVS and CSOPVS and prior evidence linking CSOPVS to CSVD and cerebral amyloid angiopathy in particular,^12-15,22^ we found no associations between CSOPVS and future stroke risk and consistently weaker cross-sectional associations with vascular risk factors and other imaging markers than for BGPVS, notably with no evidence of an association between CSOPVS and the presence of strictly lobar CMBs. It might be that CSOPVS are more difficult to rate accurately. Although not assessed in this study, interrater reliability for CSOPVS has been reported to be lower than that for BGPVS, possibly due to disagreement regarding the rating of small or faint linear PVS, which are more common in the centrum semiovale than basal ganglia region.^25^ The multicenter, multirater design of our study may have compounded this problem, although in the 2-stage meta-analysis we undertook as a sensitivity analysis, we found no strong evidence of an association between CSOPVS and stroke risk within any individual study. Another possibility is that CSOPVS are only weakly associated with the burden of cerebrovascular disease in relatively unselected patients with ischemic stroke or TIA. The studies linking CSOPVS to cerebral amyloid angiopathy (and indirectly to ICH risk) were undertaken in intracerebral hemorrhage survivors,^12-15^ a high-risk group with advanced CSVD, whereas few participants in our study had previous ICH or multiple strictly lobar microbleeds suggestive of cerebral amyloid angiopathy. Finally, CSOPVS counts were generally higher than BGPVS counts, and it might be that the threshold of >20 CSOPVS we chose to define the highest PVS burden group was lower than the threshold at which stroke risk begins to increase. However, based on our data, patients with >40 CSOPVS are rare.

The strengths of our study include its large sample size, its multicenter international study sample (increasing generalizability), and a comprehensive assessment of CSVD markers. Pooling data allowed adjustment for multiple covariates and consideration of higher categories of PVS burden, even for ICH, which has a much lower incidence than ischemic stroke. Our results provide information on which CSVD marker might be most informative for each outcome—important in clinical practice, in which rating each available marker individually might be impractical.

Our study has several limitations. Of most importance, imaging ratings were performed locally for each study, with the potential for systematic differences in ratings between studies, especially for CSOPVS. However, all raters were trained and working in expert centers, and we attempted to mitigate this for centers that rated PVS specifically for the current analysis by providing training using a standardized manual including reference images for each category and location. Although PVS were rated using axial T2 sequences only, MRI protocols were not standardized between studies, and we lacked data on acquisition parameters such as field strength.^36^ Although the number of studies included in our analysis reduces the potential for spurious results, the possibility of systematic differences in PVS ratings does mean that associations with characteristics varying heavily between studies should be interpreted cautiously. In particular, several studies recruited only patients with AF, whereas the prevalence of AF in some others was low—although the negative association between AF and PVS burden (and other CSVD markers) we observed might also reflect the cause of the qualifying stroke or TIA.

Other limitations include lack of information on changes in, and adherence to, antithrombotic and other secondary prevention medication during follow-up (although systematic variation according to PVS burden is unlikely); incomplete data for lacune presence and cerebral atrophy; the use of mainly European and East Asian studies; a lack of data on ethnicity, which might influence stroke risk and the type and prevalence of CSVD^37,38^; and incomplete data on exact ICH location and ischemic stroke etiology, which might have provided information on mechanisms linking PVS to stroke risk. We recruited studies through a consortium established to investigate cerebral microbleeds, thereby excluding studies that did not acquire MRI sensitive to cerebral microbleeds, potentially reducing our sample size and generalizability. Although we included follow-up information to 5 years, our median duration of follow-up was 1.44 years, limiting the precision with which we could estimate longer-term associations between PVS burden and our outcomes of interest.

Our study found clinically relevant associations of BGPVS when assessed visually using a semiquantitative scale by multiple raters, without standardized MRI acquisition protocols. Although consistent with how CSVD markers are assessed in current clinical practice, automated methods for PVS measurement have recently been described,^39^ potentially allowing more objective standardized ratings and assessment of more complex parameters, such as PVS volume.^40^ By reducing measurement error and avoiding loss of information through categorization, such measurements might provide greater predictive performance than those obtained in this study. Studies using these methods would help address whether measurement difficulties contributed to the weaker associations of CSOPVS we observed, especially if combined with standardized MRI protocols. Further study of how MRI acquisition parameters influence PVS visibility would also be informative. Finally, the causality of the association between CSVD and PVS formation remains uncertain, with mainly cross-sectional evidence, and could be addressed by longitudinal studies including serial measurement of CSVD markers and PVS burden.

## Appendix Authors

**Table:**

Name	Location	Contribution
Jonathan G. Best, MD	UCL Stroke Research Centre, Department of Brain Repair and Rehabilitation, UCL Queen Square Institute of Neurology, London, United Kingdom	Drafting/revision of the article for content, including medical writing for content; drafting/revision of the manuscript for content, including medical writing for content; major role in the acquisition of data; study concept or design; and analysis or interpretation of data
Gareth Ambler, PhD	Department of Statistical Science, University College London, United Kingdom	Drafting/revision of the article for content, including medical writing for content; major role in the acquisition of data; and analysis or interpretation of data
Duncan Wilson, PhD	UCL Stroke Research Centre, Department of Brain Repair and Rehabilitation, UCL Queen Square Institute of Neurology, London, United Kingdom	Drafting/revision of the article for content, including medical writing for content; major role in the acquisition of data
Houwei Du, MD	Stroke Research Centre, Department of Neurology, Fujian Medical University Union Hospital, Fuzhou, People's Republic of China	Drafting/revision of the article for content, including medical writing for content; major role in the acquisition of data
Keon-Joo Lee, MD	Department of Neurology, Korea University Guro Hospital, Seoul, Republic of Korea	Drafting/revision of the article for content, including medical writing for content; major role in the acquisition of data
Jae-Sung Lim, MD, PhD	Department of Neurology, Asan Medical Center, University of Ulsan College of Medicine, Seoul, Republic of Korea	Drafting/revision of the article for content, including medical writing for content; major role in the acquisition of data
Kay Cheong Teo, MBBS, MRCP	Division of Neurology, Department of Medicine, The University of Hong Kong	Drafting/revision of the article for content, including medical writing for content; major role in the acquisition of data
Henry Mak, MD	Department of Diagnostic Radiology, The University of Hong Kong	Drafting/revision of the article for content, including medical writing for content; major role in the acquisition of data
Young Dae Kim, MD, PhD	Department of Neurology, Yonsei University College of Medicine, Seoul, Republic of Korea	Drafting/revision of the article for content, including medical writing for content; major role in the acquisition of data
Tae-Jin Song, MD, PhD	Department of Neurology, Seoul Hospital, Ewha Womans University College of Medicine, Seoul, Republic of Korea	Drafting/revision of the article for content, including medical writing for content; major role in the acquisition of data
Derya Selcuk Demirelli, MD	Department of Neurology, Sisli Hamidiye Etfal Teaching and Research Hospital, University of Health Sciences, Turkey	Drafting/revision of the article for content, including medical writing for content; major role in the acquisition of data
Masashi Nishihara, MD	Department of Radiology, Saga University Faculty of Medicine, Japan	Drafting/revision of the article for content, including medical writing for content; major role in the acquisition of data
Masaaki Yoshikaw, MD	Division of Neurology, Department of Internal Medicine, Saga University Faculty of Medicine, Japan	Drafting/revision of the article for content, including medical writing for content; major role in the acquisition of data
Marta Kubacka, MD	Stroke Center Klinik Hirslanden Zürich; University of Basel, Switzerland	Drafting/revision of the article for content, including medical writing for content; major role in the acquisition of data
Annaelle Zietz, MMed	Department of Neurology and Stroke Center University Hospital and University of Basel, Switzerland	Drafting/revision of the article for content, including medical writing for content; major role in the acquisition of data
Rustam Al-Shahi Salman, MD, PhD	Centre for Clinical Brain Sciences, School of Clinical Sciences, University of Edinburgh, United Kingdom	Drafting/revision of the article for content, including medical writing for content; major role in the acquisition of data
Hans Rolf Jäger, MD	Neuroradiological Academic Unit, Department of Brain Repair and Rehabilitation, UCL Queen Square Institute of Neurology, University College London, United Kingdom	Drafting/revision of the article for content, including medical writing for content; major role in the acquisition of data
Gregory Y.H. Lip, MD	Liverpool Centre for Cardiovascular Science at University of Liverpool, Liverpool John Moores University and Liverpool Heart & Chest Hospital, United Kingdom	Drafting/revision of the article for content, including medical writing for content; major role in the acquisition of data
Leonidas Panos, MD	Department of Neurology, Inselspital, Bern University Hospital, Switzerland	Drafting/revision of the article for content, including medical writing for content; major role in the acquisition of data
Martina B. Goeldlin, MD	Department of Neurology, Inselspital, Bern University Hospital, Switzerland	Drafting/revision of the article for content, including medical writing for content; major role in the acquisition of data
Lee-Anne Slater, MBBS, FRANZCR, MMed	Department of Imaging, School of Clinical Sciences at Monash Health, Monash University, Melbourne, Australia	Drafting/revision of the article for content, including medical writing for content; major role in the acquisition of data
Christopher Charles Karayiannis, MD	Peninsula Clinical School, Peninsula Health, Monash University, Melbourne, Australia	Drafting/revision of the article for content, including medical writing for content; major role in the acquisition of data
Thanh G. Phan, MBBS, FRACP, PhD	Stroke and Ageing Research Group, School of Clinical Sciences at Monash Health, Monash University, Melbourne, Australia	Drafting/revision of the article for content, including medical writing for content; major role in the acquisition of data
Maximilian Bellut, MD	Department of Neurology, University Hospital of Würzburg, Würzburg, Germany	Drafting/revision of the article for content, including medical writing for content; major role in the acquisition of data
Jill Abrigo, MD	Department of Imaging and Interventional Radiology, Prince of Wales Hospital, The Chinese University of Hong Kong	Drafting/revision of the article for content, including medical writing for content; major role in the acquisition of data
Cyrus Cheng, MD	Division of Neurology, Department of Medicine and Therapeutics, Prince of Wales Hospital, The Chinese University of Hong Kong	Drafting/revision of the article for content, including medical writing for content; major role in the acquisition of data
Thomas W. Leung, MD	Division of Neurology, Department of Medicine and Therapeutics, Prince of Wales Hospital, The Chinese University of Hong Kong	Drafting/revision of the article for content, including medical writing for content; major role in the acquisition of data
Winnie Chu, MD	Department of Imaging and Interventional Radiology, Prince of Wales Hospital, The Chinese University of Hong Kong	Drafting/revision of the article for content, including medical writing for content; major role in the acquisition of data
Francesca Chappell, PhD	Centre for Clinical Brain Sciences, School of Clinical Sciences, University of Edinburgh, United Kingdom	Drafting/revision of the article for content, including medical writing for content; major role in the acquisition of data
Stephen D.J. Makin, PhD	Institute of Applied Health Sciences, University of Aberdeen, United Kingdom	Drafting/revision of the article for content, including medical writing for content; major role in the acquisition of data
Dianne H.K. van Dam-Nolen, MD	Department of Radiology and Nuclear Medicine, Erasmus University Medical Centre, Rotterdam, The Netherlands	Drafting/revision of the article for content, including medical writing for content; major role in the acquisition of data
M. Eline Kooi, PhD	Department of Radiology and Nuclear Medicine, CARIM School for Cardiovascular Diseases, Maastricht University Medical Centre, The Netherlands	Drafting/revision of the article for content, including medical writing for content; major role in the acquisition of data
Sebastian Köhler, PhD	Department of Psychiatry and Neuropsychology, School for Mental Health and Neuroscience, Maastricht University, The Netherlands	Drafting/revision of the article for content, including medical writing for content; major role in the acquisition of data
Julie Staals, MD, PhD	Department of Neurology and Cardiovascular Research Institute Maastricht (CARIM), Maastricht University Medical Centre, The Netherlands	Drafting/revision of the article for content, including medical writing for content; major role in the acquisition of data
Grégory Kuchcinski, MD, PhD	University of Lille, Inserm, CHU de Lille, Lille Neuroscience & Cognition, France	Drafting/revision of the article for content, including medical writing for content; major role in the acquisition of data
Régis Bordet, MD	University of Lille, Inserm, CHU de Lille, Lille Neuroscience & Cognition, France	Drafting/revision of the article for content, including medical writing for content; major role in the acquisition of data
Florian Dubost, PhD	Biomedical Imaging Group Rotterdam, Department of Radiology and Nuclear Medicine, Erasmus University Medical Centre, Rotterdam, The Netherlands	Drafting/revision of the article for content, including medical writing for content; major role in the acquisition of data
Joanna M. Wardlaw, MD	Centre for Clinical Brain Sciences, School of Clinical Sciences, University of Edinburgh, United Kingdom	Drafting/revision of the article for content, including medical writing for content; major role in the acquisition of data
Yannie O.Y. Soo, MD	Division of Neurology, Department of Medicine and Therapeutics, Prince of Wales Hospital, The Chinese University of Hong Kong	Drafting/revision of the article for content, including medical writing for content; major role in the acquisition of data
Felix Fluri, MD	Department of Neurology, University Hospital of Würzburg, Germany	Drafting/revision of the article for content, including medical writing for content; major role in the acquisition of data
Velandai K. Srikanth, MD, PhD	National Centre for Healthy Ageing; Peninsula Clinical School, Central Clinical School, Monash University, Melbourne, Australia	Drafting/revision of the article for content, including medical writing for content; major role in the acquisition of data
Simon Jung, MD	Department of Neurology, Inselspital, Bern University Hospital, Switzerland	Drafting/revision of the article for content, including medical writing for content; major role in the acquisition of data
Nils Peters, MD	Stroke Center Klinik Hirslanden Zürich, Switzerland	Drafting/revision of the article for content, including medical writing for content; major role in the acquisition of data
Hideo Hara, PhD	Division of Neurology, Department of Internal Medicine, Saga University Faculty of Medicine, Japan	Drafting/revision of the article for content, including medical writing for content; major role in the acquisition of data
Yusuke Yakushiji, PhD	Department of Neurology, Kansai Medical University, Osaka, Japan	Drafting/revision of the article for content, including medical writing for content; major role in the acquisition of data
Dilek Necioglu Orken, MD	Department of Neurology, Istanbul Arel University, Turkey	Drafting/revision of the article for content, including medical writing for content; major role in the acquisition of data
Ji-Hoe Heo, MD	Department of Neurology, Yonsei University College of Medicine, Seoul, Republic of Korea	Drafting/revision of the article for content, including medical writing for content; major role in the acquisition of data
Gary Kui Kai Lau, DPhil	Division of Neurology, Department of Medicine, The University of Hong Kong	Drafting/revision of the article for content, including medical writing for content; major role in the acquisition of data
Hee-Joon Bae, MD	Department of Neurology, Seoul National University Bundang Hospital, Seoul National University College of Medicine, Seongnam, Republic of Korea	Drafting/revision of the article for content, including medical writing for content; major role in the acquisition of data
David J. Werring, PhD	UCL Stroke Research Centre, Department of Brain Repair and Rehabilitation, UCL Queen Square Institute of Neurology, London, United Kingdom	Drafting/revision of the article for content, including medical writing for content; major role in the acquisition of data; study concept or design; and analysis or interpretation of data

## Glossary

AF: atrial fibrillation
ARWMC: age-related white matter changes
BGPVS: basal ganglia enlarged perivascular spaces
CSOPVS: centrum semiovale enlarged perivascular spaces
CSVD: cerebral small vessel disease
ICH: intracranial hemorrhage
IQR: interquartile range
MICON: Microbleeds International Collaborative Network
PVS: visible perivascular spaces
TIA: transient ischemic attack
WMH: white matter hyperintensities
